# Analysis of Post-COVID-19 Guillain–Barré Syndrome over a Period of One Year in the University Hospital of Split (Croatia)

**DOI:** 10.3390/neurolint15040086

**Published:** 2023-11-06

**Authors:** Niko Dunkić, Marija Nazlić, Valerija Dunkić, Ivica Bilić

**Affiliations:** 1 Practice of General Medicine, A.G. Matoša 2, 21000 Split, Croatia; nikodunkic97@gmail.com; 2 Faculty of Science, University of Split, Ruđera Boškovića 33, 21000 Split, Croatia; mnazlic@pmfst.hr (M.N.); dunkic@pmfst.hr (V.D.); 3 School of Medicine, University of Split, Šoltanska 2, 21000 Split, Croatia; 4 Neurology Department, University Hospital Split, Spinčićeva 1, 21000 Split, Croatia

**Keywords:** Guillain–Barré syndrome, immune response, neurotropism, SARS-CoV-2

## Abstract

The virus SARS-CoV-2 (Severe Acute Respiratory Syndrome Coronavirus 2) is capable of attacking the nervous system in several ways and leading to neurological diseases such as GBS (Guillain–Barré syndrome) through the resulting neurotropism and immune response. The aim of this study is to show the relationship between Coronavirus disease (COVID-19) and GBS and to better understand the clinical symptoms to prevent poor outcomes. Data from 15 patients were extracted from the Department of Neurology, University Hospital of Split, Croatia, for the year 2021. The age of the patients ranged from 26 to 89 years, of whom 27% were women. Sixty seven percent of all GBS patients recovered from COVID-19 infection, whereas post-vaccinal polyradiculoneuritis was detected in 6%. Forty four percent of the patients who developed GBS had a severe form of COVID-19 infection. Forty percent of patients were treated with intravenous immunoglobulins (IVIG), followed by therapeutic plasma exchange (PLEX) in 27%. After the therapy, improvement was observed in 13 patients, while two patients died. The results suggest that SARS-CoV-2 triggers GBS because it follows a similar pattern of infection as the other viral and bacterial agents that contribute to the onset of GBS. There is no evidence that prior infection with COVID-19 worsens the clinical presentation of GBS.

## 1. Introduction

Guillain–Barré syndrome (GBS) is an acute inflammatory and usually demyelinating polyradiculoneuropathy first described in 1916 by Georges Guillain, Jean Alexandre Barré, and Andre Strohl [1]. It occurs due to an autoimmune reaction called molecular mimicry. It is usually triggered by a previous infection resulting in the formation of anti-ganglioside antibodies that attack axonal membrane proteins, which leads to ascending symmetric weakness of the limbs, sensory disturbances, and the absence of or reduction in deep tendon reflexes. The incidence of GBS ranges from 0.81 to 1.91 cases per 100,000 people in a one-year period (median 1.11), and after the eradication of poliomyelitis, it is the most common cause of flaccid paralysis worldwide [2]. GBS can be divided into a few subtypes. The most common is acute inflammatory demyelinating polyradiculoneuropathy (AIDP). Acute motor axonal neuropathy (AMAN) and acute motor sensory axonal neuropathy (AMSAN) are associated with a worse prognosis. The rarer Miller–Fisher syndrome (MFS) is a subtype of GBS defined by a triad of clinical features including ophthalmoplegia, areflexia, and ataxia [3]. Bickerstaff brainstem encephalitis (BBE) manifests similarly to MFS with the additional finding of impaired consciousness due to extension of immune attack to the reticular formation of the pons. Less common variants include pharyngeal cervical–brachial weakness and pandysautonomia [4].

The male dominance in GBS is inexplicable and differs from the female dominance seen in most classic autoimmune diseases [5]. Most patients with GBS have a previous event that occurred within 4 weeks before the onset of neurologic symptoms. The most common is infection with *Campylobacter jejuni*, which occurs in 25–50% of adult patients. Among other infections, the association between GBS and coronavirus is now increasingly being investigated. Recent evidence suggests that SARS-CoV-2 is a trigger for GBS because it follows a similar parainfectious pattern as the other viral agents that contribute to the onset of GBS [6,7].

GBS was also associated with certain vaccinations and the use of checkpoint inhibitors in oncology [8,9]. Nerve conduction test and cerebrospinal fluid (CSF) findings with albuminocytologic dissociation are important for diagnosis, although both tests may be within reference values in the early stages. To date, the only accepted therapeutic methods are therapeutic plasma exchange and intravenous administration of immunoglobulins. Plasma exchange treatment has been found to provide optimal results when given within two to four weeks of the first signs of weakness [10]. However, it remains a very serious disease that is fatal in approximately 5% of affected individuals despite treatment, up to 20% of survivors are unable to walk independently within the first year, and as many as a quarter of patients require artificial respiration [2,9].

At the end of 2019, a new strain of the virus labeled as SARS-CoV-2 appeared in the city of Wuhan. Due to the ease of transmission, the whole world soon underwent an unparalleled pandemic [11]. The main manifestation of COVID-19 is interstitial pneumonia, which can rapidly lead to respiratory failure [12]. From the first set of cases registered in China from 31 December 2019 to 28 April 2020, at the international level, more than 3,000,000 cases were reported in more than 185 countries, with 207,265 deaths [13].

An important finding was that SARS-CoV-2 enters the host cells through angiotensin-2 converting enzyme (ACE2), which is expressed in type II pneumocytes, cardiomyocytes, vascular endothelium, smooth muscle cells, enterocytes, and neuro cells. It can affect both the peripheral and central nerve system [14]. It is still uncertain how the coronavirus causes various neurological symptoms and what the mode of entry is. Several possible mechanisms have been proposed [15]. 

The virus is thought to enter the central nervous system (CNS) by hematogenous spread or retrograde dissemination. The first mechanism is based on the existence of the enzyme ACE as a functional receptor for SARS-CoV-2 through which it enters. SARS-CoV-2 can further infect leukocytes, which then cross the blood–brain barrier via a mechanism known as the Trojan horse or enter the choroid plexus via the blood–CSF barrier. The second neuropathological pathway describes retrograde invasion via peripheral or cranial nerves and entry into the brain via the olfactory bulb, through which SARS-CoV-2 spreads transneuronal to different brain regions. Apart from which route of transmission the virus uses after entering the CNS, neurotropism and the subsequent immune response result in CNS pathology leading to disease [16]. Now, the possibility of the virus entering through the vagus and trigeminal nerve is also being considered [17].

The aim of this study was to provide insight into the predisposing factors, to classify patients suffering from GBS according to age groups and sex, to record the total number of GBS patients throughout 2021, and to compare it with prepandemic data at the Split–Dalmatia County level (Croatia). The aim was to show the percentage at which infection with COVID-19 preceded the onset of GBS and the time interval between infection with COVID-19 and the onset of GBS. The aim was also to classify patients according to the month of hospital admission and to compare these data with current epidemiological data on COVID-19 infections and to determine whether patients who had previously recovered from COVID-19 stayed longer in the hospital because of the infection, which therapy was prescribed most frequently, and what the condition was at hospital discharge.

Based on the evidence to date, this article was written to contribute to the understanding of the relationship between COVID-19 and GBS and to provide a better understanding of the clinical symptoms to prevent poor outcomes.

## 2. Materials and Methods

The data were obtained by querying the central computer of the Department of Neurology with the code ICD 10. (International Classification of Diseases) G 61.0 from the archives of the Department of Neurology, University Hospital Split, Croatia, in 2021. The collected data were entered into the program Microsoft Excel for data processing and are presented in the word processing program Microsoft Word.

Statistical data processing was performed using the GraphPad Prism 9 statistical program and Microsoft Excel (Microsoft Office Professional Plus 2019). Categorical variables of descriptive statistics are presented as whole numbers and percentages. Differences between the two groups with respect to continuous variables were analyzed by analysis of variance (ANOVA) with the post hoc Tukey HSD test.

The following parameters were recorded for each patient: age, sex, date of admission to the neurology clinic, length of hospital stay, presence of previous infection COVID-19 and its association with GBS, and length of hospital stay, therapy used, and neurological status at hospital discharge.

## 3. Results

This study includes patients who were treated at a University Hospital in Split (Croatia), with a total of 15 patients with a confirmed diagnosis of Guillain–Barré syndrome (GBS) throughout 2021.

### 3.1. Diagnostic Criteria

Fifteen patients were enrolled in this study. For diagnostic criteria, we used the Brighton Collaboration criteria. According to those criteria, a large number of our 15 patients belonged to group 1 (highest probability of GBS). All of them had a monophasic and progressive disease course. At baseline, they had bilateral and flaccid lower limb weakness and decreased or absent deep tendon reflexes, which showed a relatively smooth ascending course with upper limb involvement, and some of them also had cranial nerve involvement. In addition to clinical features, we also performed nerve conduction studies and cerebrospinal fluid analysis. In the nerve conduction studies, 13 of 15 patients had signs of sensory polyneuropathy, and five of them showed an axonal form of the syndrome (AMAN). AMAN included axonal loss with reduced compound muscle action potentials (CMAP) and motor conduction block (CB) with or without subsequent reversible conduction interruption. Eight of them had the demyelinating subtype, which showed prolonged distal motor latency, reduced conduction velocity, and increased temporal dispersion and reduced conduction velocities. CSF examination revealed increased protein counts with normal cell numbers (albumin cytologic dissociation) in all of them.

### 3.2. Age and Sex Distribution of Patients with Guillain–Barré Syndrome

**Figure 1:**
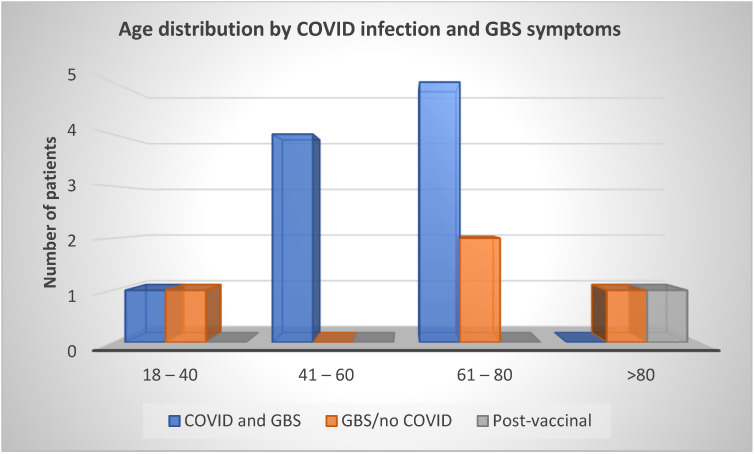
Comparison of the age of patients who had a COVID-19 infection and consequently GBS and those who had GBS and were not previously infected with the coronavirus.

According to Figure 1, taking into account the age of patients with GBS, it is noted that the volume of the syndrome was preceded by a COVID-19 infection mainly in the working age population. As the age of patients increased, the number of GBS cases without confirmed COVID-19 also increased, especially in patients over eighty years old.

**Figure 2:**
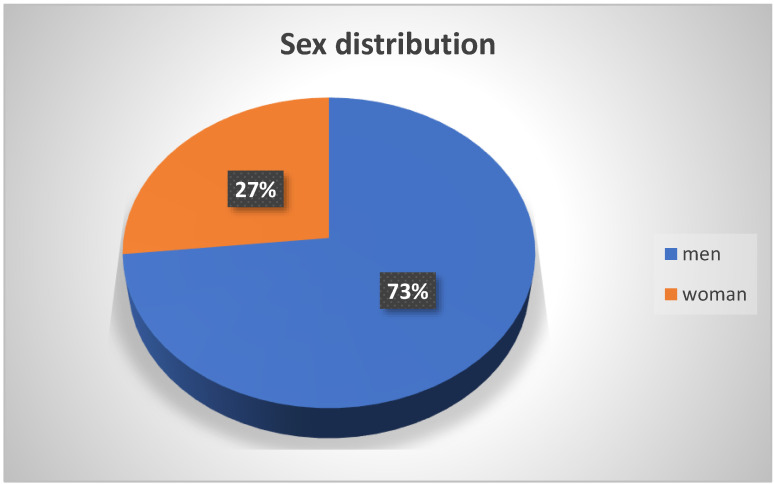
Distribution of GBS patients by sex.

Of the total number of patients observed, 73% were male, while females accounted for 27% (Figure 2).

### 3.3. Acute Polyradiculoneuritis and COVID-19 Infection

**Figure 3:**
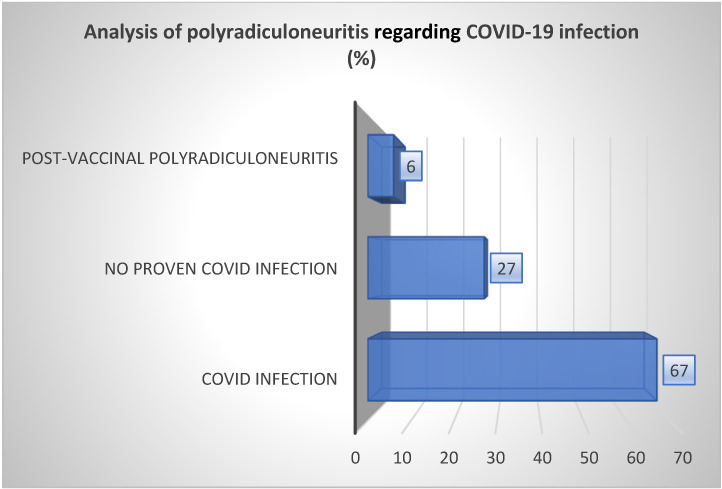
Analysis of acute polyradiculoneuritis in relation to a previously healed COVID-19 infection.

Of the total number of GBS patients, 67% had recovered from COVID-19 infection, no SARS-CoV-2 virus was isolated in 27% of them, and post-vaccinal polyradiculoneuritis was detected in 6% of patients (1 patient) (Figure 3).

### 3.4. The Time Interval between Overcoming the COVID-19 Infection and the Appearance of the First Symptoms of GBS

**Figure 4:**
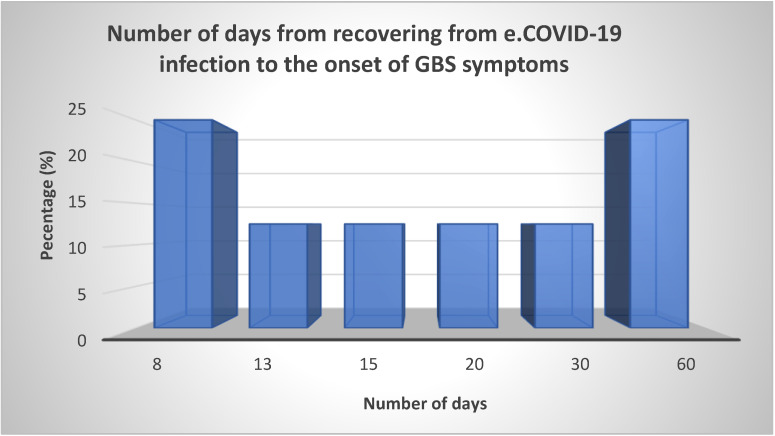
Illustration of the period from overcoming the COVID-19 infection to the appearance of GBS symptoms.

The time interval from overcoming COVID-19 infection to the appearance of GBS symptoms ranged from 8 to 60 days. Twenty five percent of patients developed GBS 8 days after infection with COVID, and the same number of GBS patients were ill after 60 days. The other 50% of patients became ill within this 8- to 60-day period (Figure 4).

### 3.5. Monthly Presentation of Admissions of Patients in Addiction with Epidemiological Data of COVID-19 Infections

**Figure 5:**
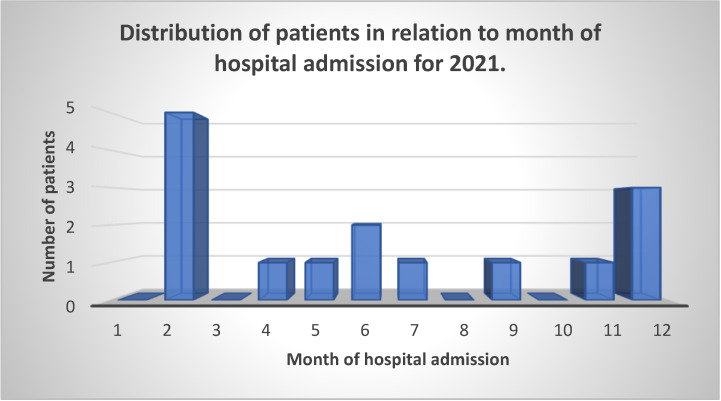
Illustration of the distribution of patients in relation to the month of admission to Split University Hospital due to GBS symptoms for 2021.

**Figure 6:**
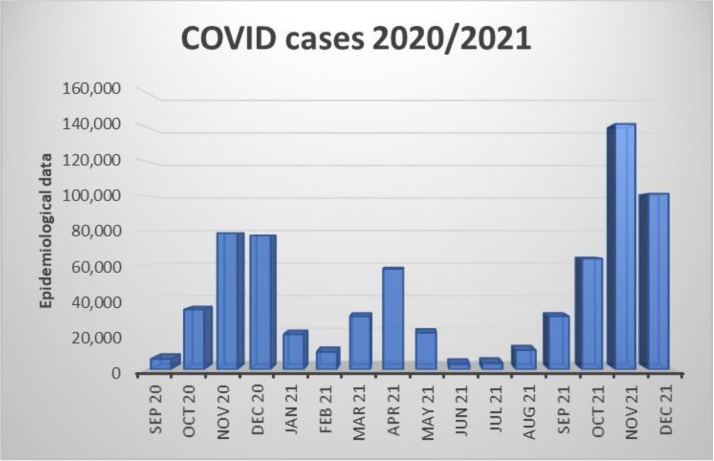
Patients with COVID-19 infection by month, from September 2020 to December 2021 [18].

Figure 5 shows that the winter months dominate in the number of GBS patients, and two months, February and December, are particularly highlighted. These data show that the number of individuals with COVID-19 increases during the colder months of the year, with a particularly large increase in the number of patients in November and December in 2020 and 2021 (Figure 6).

### 3.6. Duration and Form of Hospitalization

**Figure 7:**
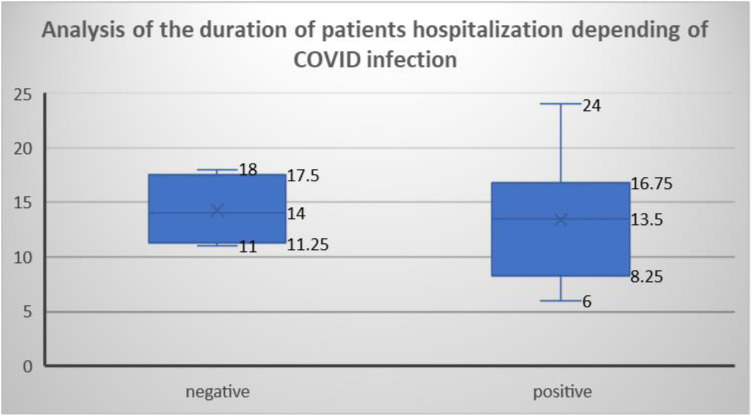
ANOVA analysis of the duration of hospitalization of patients in whom GBS was not triggered by COVID-19 infection and of patients who developed GBS after COVID-19 infection.

Using an ANOVA analysis of the length of hospital stay of patients with GBS depending on whether or not this syndrome was preceded by COVID-19 infection, we obtained that the COVID-19-negative patients were treated for an average of 14 days, and patients with proven previous COVID-19 infection were treated for 13.5 days. The 95% confidence interval limits for COVID-19-negative patients ranged from 11.25 to 17.5 and for positive patients from 8.25 to 16.75. No significant difference in length of hospital stay was found between these patients, as their ranges reliably overlapped (Figure 7).

**Figure 8:**
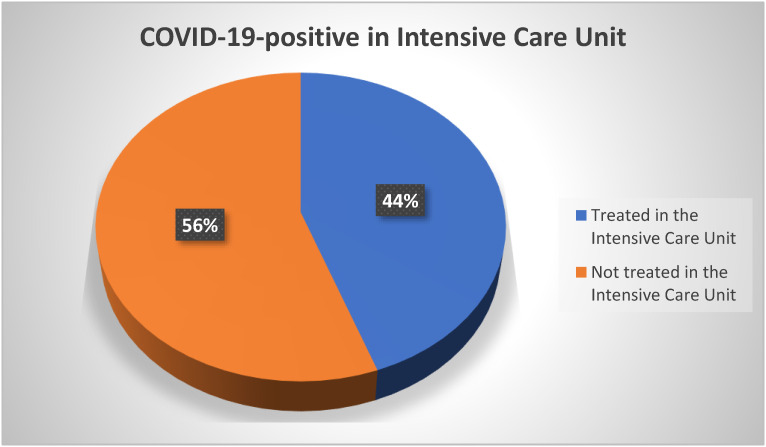
Presentation of patients treated in the Intensive Care Unit due to COVID-19 infection.

In total, 44% of patients who developed COVID-19 infection and subsequently GBS had a severe form of COVID-19 infection that required intensive ventilatory support in treatment units (Figure 8).

### 3.7. Therapy and Treatment Outcome 

In our study, most patients, namely 50%, received intravenous therapy with immunoglobulins (IVIG), and 37% received therapeutic plasma exchange (PLEX), whereas a combination of these two therapies was much less common (13%). After the applied therapy, improvement was observed in 13 of 15 patients, while two patients died. 

## 4. Discussion

Fifteen patients were enrolled in this study. For diagnostic criteria, we used the Brighton Collaboration criteria. According to those criteria, a large number of our 15 patients belonged to group 1 (highest probability of GBS) [19].

The aim of this study was to classify patients suffering from GBS according to age groups and sex, to record the total number of GBS patients in 2021 and compare it with the prepandemic data at the Split–Dalmatia County (Croatia) level, to show how the total number of GBS cases has increased with Coronavirus disease (COVID-19), and to emphasize that moderate or even severe COVID-19 infection leads to GBS.

Data were collected by reviewing the archives of Split University Hospital. The youngest patient included in this study was 26 years old, and the oldest was 89 years old. Looking at the distribution of the total number of patients by age, divided into age intervals of 20 years each, the largest proportion of GBS patients was between 61 and 80 years old, accounting for 47% of the total number of patients. This finding is consistent with studies in the USA. In the U.S. studies, the largest proportion of GBS patients is found in the elderly population, with an incidence of 8.61 per 100,000 people aged 70–79 years [20]. The proportion of patients aged 18–40 years was 6%, it was 27% in the age group 41–60 years, and the proportion of patients older than 80 years was 20%.

In 2019, the global point prevalence of GBS (per 100,000) showed a broad increase with age. More specifically, the total number of cases was highest in the 5–9 year age group but then declined to the 25–29 year age group and then increased to the 60–64 year age group before decreasing again in the oldest age group [21]. 

People who suffered COVID-19 infection and later GBS were mainly the working-age population, and as patients aged, more and more cases of GBS occurred without confirmed COVID-19 infection, especially in the group over 80 years of age (Figure 1). The claim that COVID-19 infection is less common in the elderly over eighty years of age is consistent with worldwide epidemiologic data, likely due to less socialization [22]. Considering the distribution of the total number of patients in our study by sex, 73% of the affected patients were male, and only 27% of women were affected by this syndrome, supporting the theory that GBS is one of the rare autoimmune diseases that affects men more frequently (Figure 2) [4]. In one study, men were 2.3 times more likely to be affected than women, and they were also more likely to have a poorer response to immunomodulatory IVIG therapy [23]. 

Seventy-three percent of men affected by GBS had previously been infected with the SARS-CoV-2 virus, whereas this figure was slightly lower for women, at 50% [24]. Possible explanations for male predominance may include differences in viral exposure, smoking, differences in the chromosomal expression of ACE2, expression of ACE2 in testicular tissue, regulation of the immune system by sex hormones, or sex differences in RAAS regulation [25]. Estradiol, a hormone found at high levels in women, increases the level of soluble ACE2, which blocks the entry of SARS-CoV-2 into cells, through its effect on proteins expressed in the lung [26]. Also, it has been suggested that males have an increased susceptibility to binding of the SARS-CoV-2 spike (S) glycoprotein and angiotensin-converting enzyme 2 (ACE2) receptors on host cells, resulting in downregulation of ACE2 [27].

According to the latest census of 2021, a total of 425,412 people live in the Republic of Croatia in the area of Split–Dalmatia County [28]. The total number of GBS patients, according to the archival data of University Hospital Split for the year 2021 was fifteen people. The average incidence in Split–Dalmatia County was 3.5 per 100,000 population, which is still a higher incidence than in the five-year period before the pandemic from 2012 to 2016, where the average incidence was 2.37 patients per 100,000 population [29]. Considering that the mean worldwide incidence of GBS is 1.11 per 100,000 population, the figure of 3.5 cases per 100,000 does not seem low and represents a 31.7 percent increase. This means that during infection with COVID-19, the likelihood of severe neurological disease such as GBS increased by almost 1/3 [21].

There are many possible reasons why this number has increased. One possible reason is that 42,415 people became ill with influenza viruses in 2012, more than in other years, which also suggests that viruses cause GBS [30]. However, the five-year average has decreased overall, particularly in 2014, when only seven patients with GBS were recorded, and in 2016, 10 were recorded [29]. When considering the increase in incidence, it should also be taken into account that in the part of the population living in the border municipalities and districts of the neighboring country Bosnia and Herzegovina, which is usually treated in the Split hospital, a large number of COVID-19 infections were recorded.

Also, from the last population list there is a significant decrease in the population in Split–Dalmatia County, so the relative number of GBS infections could be higher than in previous years [31].

In our study, 67% of the total number of GBS patients recovered from COVID-19 infection, no SARS-CoV-2 virus was isolated in 27% of them, and post-vaccinal polyradiculoneuritis was detected in 6% of patients (1 patient) (Figure 3).

Figure 5 and Figure 6 show the months in which patients were admitted to the clinical hospital center in Split and the status of the COVID-19 infection at the level of the Republic of Croatia from September 2020 to December 2021 [18]. We can note that most hospitalizations occurred in the winter months, especially in February and December, and if we take into account that the time from COVID-19 infection to the appearance of GBS was on average between 8 and 60 days (Figure 4), we can easily argue that GBS belongs to one of the post-COVID-19 syndromes. Also, in the prepandemic period, patients were more frequently hospitalized during the summer months [29]. An Italian study also found an increased incidence of GBS in northern Italy between March and April 2020 compared with the same period in 2019, suggesting a possible link between the COVID-19 pandemic wave and GBS [32].

The shortest hospital stay lasted 6 days and the longest 26 days (Figure 7). The ANOVA analysis of the length of hospital stay of patients with GBS according to whether or not the syndrome was preceded by COVID-19 infection found that the COVID-19-negative patients were treated for an average of 14 days, and patients with proven previous COVID-19 infection were treated for 13.5 days. The limits of the 95% confidence interval for the COVID-19-negative patients ranged from 11.25 to 17.5 days, and for COVID-19-positive patients, it ranged from 8.25 to 16.75. No significant difference in the length of hospital stay was found between these patients because their confidence intervals overlapped. The Spanish study supports these data, as the average length of hospital stay was 8–15 days [33]. In another study, it was found that there was no significant difference between the symptoms of patients who contracted COVID-19 and consequently GBS and those who received GBS without prior infection with SARS-CoV-2 virus [34]. This same study described that more than one-third of patients with COVID-19 infection had the most severe form of COVID-19 infection, who required mechanical ventilation [34]. In the University Hospital of Split, the percentage of such patients was 44% (Figure 8). Interestingly, almost all the patients who suffered COVID-19 had lung involvement with signs of interstitial pneumonia and needed oxygen therapy, suggesting that GBS mainly develops following moderate or severe COVID-19 infections and seems less likely in asymptomatic forms [32]. 

In our study, most patients, namely 50%, received intravenous therapy with immunoglobulins (IVIG) and 37% received therapeutic plasma exchange (PLEX), whereas a combination of these two therapies is much less common (13%). Worldwide, almost all patients with GBS were treated with IVIG, which is associated with thromboembolic side effects and can lead to prothrombotic states when combined with the SARS-CoV-2 virus, but no reports have described such side effects [34]. In a randomized controlled trial, daily administration of IVIG for five consecutive days was as effective as five plasma sessions started within 14 days. IVIG acts by inhibiting the binding of autoantibodies to GQ1b and also complements activation by anti-GQ1b in a mouse model. IVIG is preferred to plasma exchange in young children because it can be technically difficult and also in patients with cardiovascular instability because large volume shifts occur during plasma exchange [35].

The results of our study show that after the applied therapy, improvement was observed in 13 of 15 patients, whereas two patients died (13%). This is still more than one study in which 5.8% of the patients died [36]. 

It is also important to note that these two decedents had a more severe form of GBS, the AMAN form; so, we need to be even more careful in the clinical approach when a person has this subtype of GBS [3]. 

However, the question remains whether these patients died from GBS or from other concomitant diseases. From the medical history in our study, one deceased person had malignant disease, while the other was transferred to the neurology clinic after needing mechanical ventilation due to severe COVID-19 infection.

Today, many papers recommend vaccinating people with inflammatory neuropathy in any case because they have a higher risk of developing a severe form of COVID-19, the risks of vaccination alone are much lower, and the side effects are not greater in these patients than in the rest of the population. It is also important to note that women under 30 years of age had the most side effects after vaccination in the form of local pain, headache, myalgias, etc. [37]. In our work, one woman developed GBS after vaccination. She developed a mild form of AIDP and was quickly discharged home, but she had systemic lupus erythematosus, and who knows what would have happened if she had contracted COVID-19 without vaccination. 

## 5. Conclusions

The majority of patients with GBS and COVID-19 included in this study were male and between 61 and 80 years of age. Although there were more GBS patients who also had COVID-19 infection, and a large number had the most severe form of infection, no significant difference was found in the length of hospital stay between COVID-19-positive and COVID-19-negative GBS patients. 

Neurologists need to pay special attention and increased observation to people with the proven AMAN subtype, because the clinical picture is more severe, and the likelihood of death is higher. They must also keep in mind when administering the most common IVIG therapy to men that its immunomodulatory effect may not be effective.

To this day, the virus is still among us causing great harm. The total number of GBS cases is increasing, for more than 31%, and now that the pandemic is over, it is important to continue this study and try to understand better the pathophysiological relationship between SARS-CoV-2 infection and GBS. This is important to allow optimal treatment of patients suffering from this coincidence and prevent dire consequences, especially in susceptible neurological patients.

Furthermore, strategies to prevent infectious diseases and improve hygiene conditions can also be effective in reducing the prevalence of GBS.

## Data Availability

Data is contained within the article.
